# Association of Programmed Death 1 Protein Ligand (PD-L1) Expression With Prognosis in Merkel Cell Carcinoma

**DOI:** 10.3389/fmed.2020.00198

**Published:** 2020-06-05

**Authors:** Glenn J. Hanna, Alec J. Kacew, Anusha R. Tanguturi, Hans J. Grote, Victoria Vergara, Beatrice Brunkhorst, Guilherme Rabinowits, Manisha Thakuria, Nicole R. LeBoeuf, Christian Ihling, James A. DeCaprio, Jochen H. Lorch

**Affiliations:** ^1^Head and Neck Oncology, Department of Medical Oncology, Dana-Farber Cancer Institute, Boston, MA, United States; ^2^Boston University School of Medicine, Boston, MA, United States; ^3^Merck KGaA, Darmstadt, Germany; ^4^EMD Serono, Research and Development Institute, Billerica, MA, United States; ^5^Hematology/Oncology, Miami Cancer Institute, Miami, FL, United States; ^6^Dermatology, Cutaneous Oncology, Dana-Farber Cancer Institute and Brigham and Women's Hospital, Boston, MA, United States; ^7^Merck KGaA, Darmstadt, Germany; ^8^Molecular and Cellular Oncology, Department of Medical Oncology, Dana-Farber Cancer Institute, Boston, MA, United States

**Keywords:** PD-L1, merkel cell carcinoma, cancer, neuroendocrine carcinoma, prognostic biomarkers, merkel cell polyomavirus, MCPyV

## Abstract

**Background:** Merkel cell carcinoma (MCC) is a rare neuroendocrine skin cancer. Prior to the advent of immunotherapy, treatment options were limited. In our study, we evaluate the impact of tumor cell PD-L1 expression and tumor immune microenvironment on survival in MCC patients who were not treated with immune checkpoint inhibitors.

**Methods:** Clinical data and tissue samples were collected from 78 patients with confirmed MCC treated at Dana-Farber Cancer Institute. Specimens were analyzed for the distribution of PD-L1 by immunohistochemistry staining (IHC) and standardized analysis. Results were correlated with survival data.

**Results:** In this study, membrane and cytoplasmic MCC tumor cell staining for PD-L1 was detected in 22.4% (15 of 67) of cases and PD-L1 staining of intratumoral microvessels and PD-L1 positive immune cells at the infiltrative margins of the tumor in 92.5% (62 of 67) of cases. In patients untreated with immune checkpoint inhibitors, median overall survival was not different for patients based on PD-L1 expression (PD-L1+ 64 months vs. PD-L1- not reached; HR = 1.26, 95% CI: 0.46–3.45; *p* = 0.60).

**Conclusion:** PD-L1 expression is frequently detected in MCC tumor cells and tumor microenvironment. PD-L1 expression did not affect prognosis in this cohort that had not received PD-1/L1 blockade.

## Introduction

Merkel cell carcinoma (MCC) is a rare neuroendocrine skin neoplasm that resembles normal Merkel cells that reside within the basal layer of the epidermis (1). MCC typically occurs in sun-exposed areas of the head and neck and in patients with an altered immune system (2, 3). Recently, the Merkel cell polyomavirus (MCPyV) was discovered to be associated with a majority of MCC cases (4). Viral genome integration into the tumor genome precedes clonal expansion, supporting a potential pathogenic role for this virus (5). The genomic alterations seen in MCC overlap with small cell lung cancer, a more prevalent neuroendocrine carcinoma, with translocations and deletions in chromosome 1 occurring in up to 40% of cases (6).

Locally advanced MCC is typically treated with wide local excision with or without adjuvant radiotherapy, and cure rates are high (7). However, recurrence rates for MCC approach 60% with most recurrences occurring within 2 years of primary treatment−52% are reported to occur in regional nodes and 34% represent distant recurrences (8). Even in the face of locoregional recurrence, up to 60% of patients can be salvaged with re-excision and adjuvant radiotherapy (9). Factors associated with decreased overall survival are age ≥75, male sex, tumor size ≥2 cm, positive margins, and ≥1 positive node (10). Palliative chemotherapy is usually not effective beyond first-line treatment.

The advent of checkpoint blockade therapy targeting programmed cell death protein-1 (PD-1) and its ligand (PD-L1) have altered the treatment of many cancer types. Since MCC occurs more frequently in patients who are immunosuppressed (e.g., solid organ transplant patients) and since tumor and blood of patients with MCC often harbor MCPyV oncoprotein specific T-cells, MCC is a natural candidate for therapies targeting the immune system (11, 12). In recent years, three landmark immune checkpoint inhibitor studies in advanced MCC have been published. One investigated the PD-1 inhibitor pembrolizumab (13), one the PD-1 inhibitor nivolumab (14), and another the PD-L1 inhibitor avelumab (15). All studies showed impressive activity and most adverse events were low grade consisting primarily of fatigue and infusion-related reactions. Based on the avelumab JAVELIN Merkel 200 data, the FDA granted approval for avelumab as the first drug indicated for this patient population (15). Immunotherapy has now become a standard of care in this disease, having shown higher rates of durable response than expected for cytotoxic chemotherapy.

In this study, we tested whether tumoral PD-L1 expression had any impact on survival in MCC patients who had not received immunotherapy. Given that each of the immunotherapy trials did not have control arms, our data can serve as a point of comparison for patients not treated with immunotherapy (i.e., as a control group for future prospective studies). It is unlikely that a study like this will be able to be replicated in the future, as immunotherapy is now considered standard-of-care in MCC.

## Methods

Clinical data and tissue samples were analyzed from 78 patients with pathologically confirmed (CK-20 positive) MCC who were to be treated at Dana-Farber Cancer Institute, diagnosed between 2002 and 2010. The clinical data collected included age, gender, date of diagnosis, primary site of disease, clinical staging, and survival. The tissue samples analyzed consisted of cutaneous tumor biopsies and resected regional lymph node metastases. We utilized IHC with a proprietary avelumab/Merck/Pfizer analytic PD-L1 antibody clone 73-10 (a rabbit monoclonal recombinant antibody), to characterize formaldehyde fixed paraffin embedded (FFPE) tissue blocks from each patient. Individual patient samples were arranged in triplicate as a tissue microarray (TMA) to facilitate group staining.

Specimens were analyzed for the presence and distribution of PD-L1 immunoreactivity. Semi-quantitative scoring for PD-L1 utilized the H-score. The percentage of negative (0), weakly stained (1), moderately stained (2), and strongly stained (3) tumor cells was estimated. Subsequently the H-score was calculated as follows: H-score = (weak)% × 1 + (moderate)% × 2 + (strong)% × 3, as previously described (16). An H-score greater than zero was counted as positive PD-L1 staining. Two independent surgical pathologists participated in verifying the results (Ihling C and Grote HJ, Merck KGaA, Clinical Biomarkers, and Companion Diagnostics).

Given the relatively small sample size, we used general descriptive statistics to represent most data. Overall survival (OS) was defined as time from study entry to death, otherwise this was censored at date last known alive. Kaplan-Meier statistics were applied using log-rank testing to evaluate outcome data. A Pearson coefficient was used to evaluate for association. All statistical testing used a significance cutoff of <0.05 and were 2-sided.

## Results

Of the 78 cases in the population, the median age at diagnosis was 69 (range 45–95 years) as noted in Table 1. Thirty (38.5%) of 78 patients were female. The majority of patients presented with a primary site of cutaneous disease on an extremity or in the head and neck region. Twelve (15.4%) patients had stage I disease, 21 (26.9%) had stage II, 41 (52.6%) had stage III, and one (1.3%) had stage IV disease. The initial staging information for three patients was unavailable. Median survival time was 40 (range: 5–113) months. Thirty-four patients (43.5%) had died at the point of analysis.

**Table 1:** Baseline characteristics of patients with Merkel cell carcinoma.

	**(Range)**
Median age at diagnosis	69 (45–95)
Gender (%)	
Male	48 (61.5)
Female	30 (38.5)
Primary site	
Upper extremity	23 (29.5)
Lower extremity	34 (47.4)
Head & neck	19 (24.4)
Torso	1 (1.3)
Genital	1 (1.3)
Staging at diagnosis^*^	
I	12 (15.4)
II	21 (26.9)
III	41 (52.6)
IV	1 (1.3)
Mos (range) (%)	
Median survival	40 (5–113)
Death at the time of analysis (%)	34 (43.5)

* *Three patients' staging information was unavailable*.

**Figure 1:**
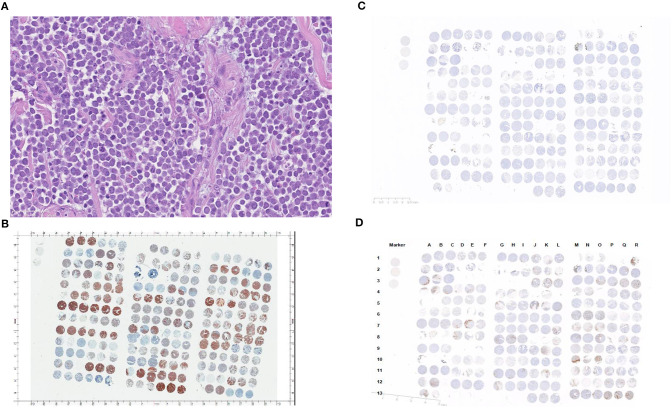
**(A)** Merkel cell carcinoma case (40x) demonstrating typical morphology with tumor cells arranged in a diffuse fashion showing scant amphophilic or eosinophilic cytoplasm and relatively uniform nuclei. The nuclei have finely dispersed chromatin without prominent nucleoli. **(B)** CK-20 staining pattern in patients with Merkel cell carcinoma (cells are arranged in groups of three, with three horizontal cells representing triplicate samples from a single tumor). **(C)** Rabbit IgG isotype immunohistochemistry (control). **(D)** PD-L1 staining in patients with Merkel cell carcinoma (cells are arranged in groups of three, with three horizontal cells representing triplicate samples from a single tumor).

Tumor samples confirmed as MCC were composed of trabecular to insular or diffuse proliferations of tumor cells with scant amphophilic or eosinophilic cytoplasm and relatively uniform nuclei sometimes exhibiting nuclear molding (Figure 1A). Additionally, CK-20 immunohistochemical staining was performed to exclude skin metastasis of small cell lung cancer (Figure 1B). Rabbit isotypes were stained for PD-L1 to serve as controls (Figure 1C). A total of 67 out of 78 cases were evaluable following immunohistochemical staining (cases were excluded if tissue washed off or <50 tumor cells were present).

PD-L1 staining in the tumor cells and in the surrounding tumor infiltrating immune cells is shown in Figure 1D. Semi-quantitative scoring for tumor cell PD-L1 using the H-score is shown in Table 2. 15 of 67 (22.4%) patient tumor samples were characterized as PD-L1 positive, with an H-score maximum of 6 for individual cases (range 1–6).

**Table 2:** Immunohistochemical staining with anti-PD-L1 clone 73-10 in patients with Merkel cell carcinoma and intratumoral expression of PD-L1.

**Subjects**	**Tumor tissue (% cells) cytoplasm**					**H-score cytoplasm**	**Tumor tissue (% cells) membrane**					**H-score membrane**	**Tumor infiltrating immune cells (TIICs) (“+” or “-”)**
	**Negative**	**Weak**	**Moderate**	**Strong**	**Sum (%)**		**Negative**	**Weak**	**Moderate**	**Strong**	**Sum (%)**		
1	97	0	3	0	100	6	97	0	3	0	100	6	-
2	98	0	2	0	100	4	98	0	2	0	100	4	-
3	97	0	3	0	100	6	97	0	3	0	100	6	-
4	98	0	2	0	100	4	98	0	2	0	100	4	+
5	99	0	1	0	100	2	99	0	1	0	100	2	+
6^*^	98	0	2	0	100	4	98	0	2	0	100	4	+
7	99	0	1	0	100	2	99	0	1	0	100	2	+
8^*^	99	1	0	0	100	1	99	1	0	0	100	1	-
9^∧^	99	0	1	0	100	2	99	0	1	0	100	2	+
10^*^	99	0	1	0	100	2	99	0	1	0	100	2	+
11	97	0	3	0	100	6	97	0	3	0	100	6	+
12	99	0	1	0	100	2	99	0	1	0	100	2	+
13	100	0	0	0	100	0	99	1	0	0	100	1	-
14	99	0	1	0	100	2	99	0	1	0	100	2	+
15	99	0	1	0	100	2	99	0	1	0	100	2	+

* *Intratumoral microvessels staining for PD-L1*.

∧ *Nuclear staining of tumor cells for PD-L1*.

*Semi-quantitative scoring for PD-L1 utilized the H-score. The percentage of negative (0), weakly stained (1), moderately stained (2) and strongly stained (3) tumor cells was estimated. Subsequently the H-score was calculated as follows: H-score = (weak)% × 1 + (moderate)% × 2 + (strong)% × 3. An H-score greater than zero was counted as positive PD-L1 staining*.

**Figure 2:**
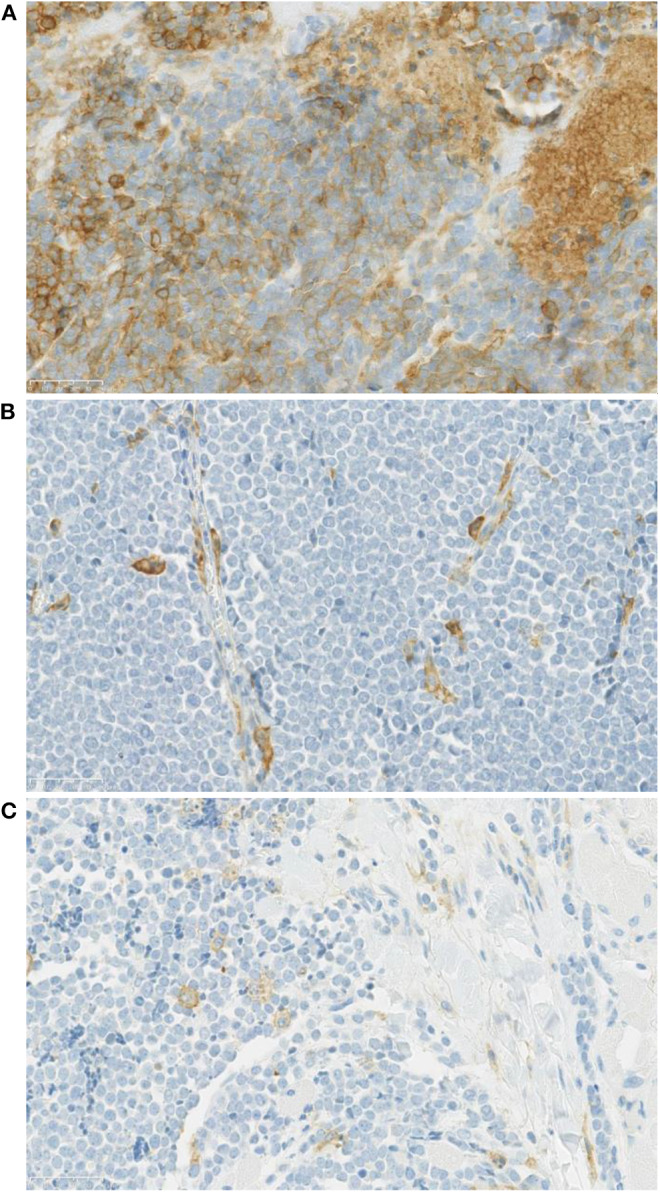
**(A)** PD-L1 staining of a Merkel cell carcinoma (case 12-5684, 40x) showing tumor cells with membrane associated as well as cytoplasmic PD-L1 staining. **(B)** A Merkel cell carcinoma sample with prominent PD-L1 staining of intra-tumoral microvessels (40x). **(C)** Tumor cells lacking PD-L1 expression. By contrast, within the tumor there are scattered PD-L1 positive immune cells with weak membrane and cytoplasmic PD-L1 staining.

**Figure 3:**
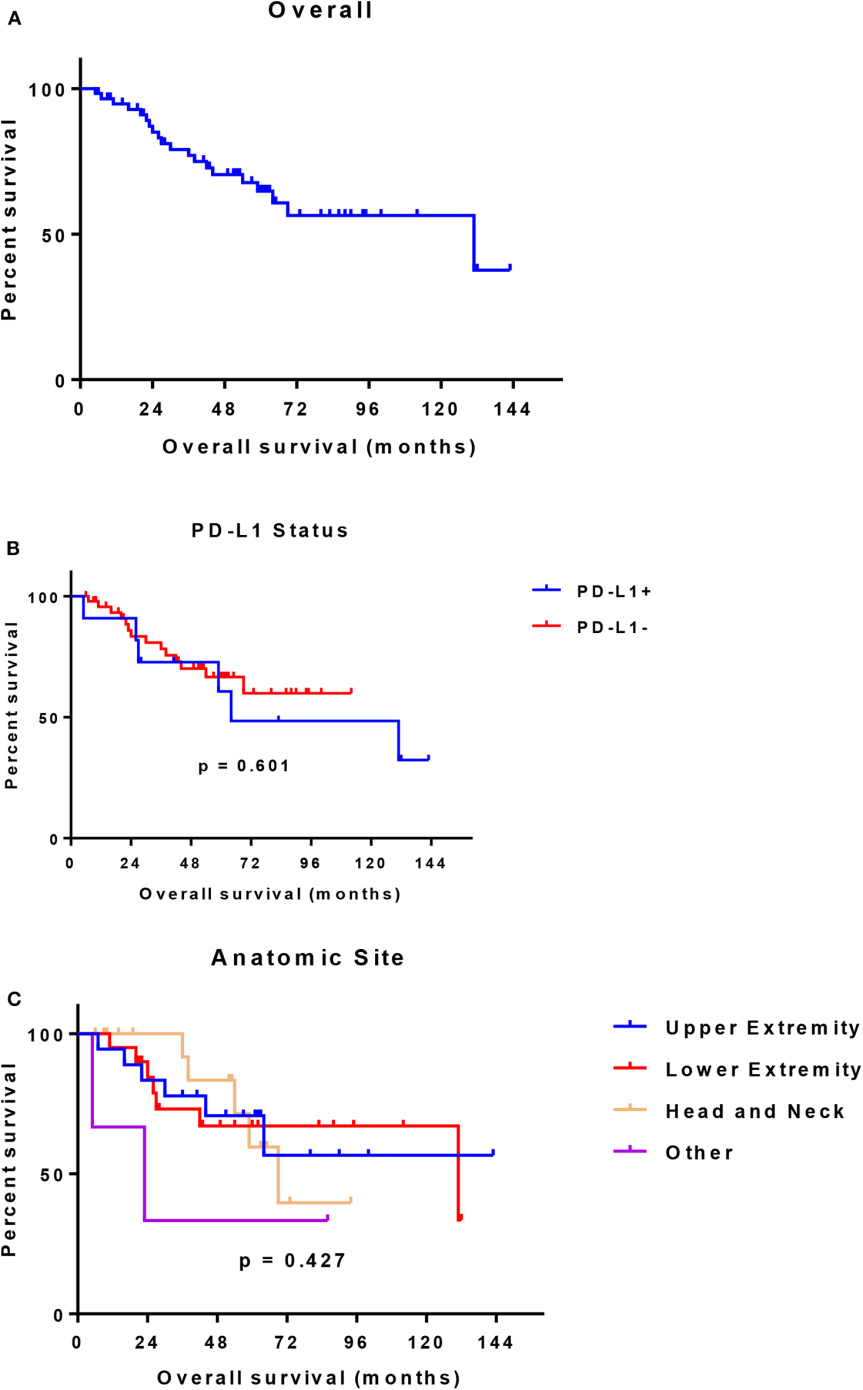
**(A)** Overall survival (all evaluable patients) **(B)** Overall survival by PD-L1 expression **(C)** Overall survival by anatomic site.

In 14 patients (20.9%), the tumor cells showed both membrane-associated staining and cytoplasmic staining for PD-L1 (Figure 2A). In several cases, prominent PD-L1 staining of intratumoral microvessels was present (Figure 2B). Immune infiltrates with PD-L1 positive cells of varying size and density were present at the infiltrative margin of the tumor in 62 (92.5%) of 67 cases. By contrast, within the tumor specimens we observed scattered PD-L1 positive immune cells but no widespread or dense immune infiltrates (Figure 2C). Neither membrane-associated nor cytoplasmic staining for PD-L1 by H-score was correlated with the presence of infiltrating immune cells (*p*-value = 0.17). Median overall survival was similar among subgroups regardless of PD-L1 expression intensity (HR 1.26, 95% CI: 0.46–3.45, *p* = 0.60) (Table 3, Figure 3) and regardless of disease site (Table 4).

**Table 3:** Median survival depending on the PD-L1 expression and stage of disease.

	**PD-L1+ expression**	**PD-L1- expression**	**Hazards ratio**	**95% CI**	***P*-value**
OS in PD-L1 expression months	64.0 months	NR	1.26^*^	0.46–3.46	0.601
Stages of the MCC disease	**OS Between Stage 1 and 2**	**OS between Stage 3 and 4**	0.99^*^	0.41–2.39	0.986
Expressed in months	69.0	NR			

NR, not reached; ^*^HR changes with the change in the follow up time period; OS, Overall survival.

**Table 4:** Median survival depending on site of disease.

	**Median OS in months**	**95% CI**	***P*-value (vs. rest of cohort)**
Upper extremity	NR	44-NR	0.43
Lower extremity	131	27-NR	0.29
Head & neck	69	38-NR	0.93
Other	5	5-NR	1.00

*CI, confidence interval; NR, not reached; OS, overall survival*.

## Discussion

MCC represents an aggressive neuroendocrine carcinoma of the skin that is characterized by high rates of locoregional recurrence and distant metastatic disease. While overall response rates of advanced disease to chemotherapy regimens are favorable, they are often of limited duration (17). The purpose of this study was to characterize tumoral PD-L1 expression patterns observed in MCC tumors and to determine if these expression patterns relate to outcomes in patients not treated with immunotherapy. Notably, three landmark studies have recently demonstrated high response rates of MCC to checkpoint blockade therapy targeting PD-1 and PD-L1 in MCC. Understanding why MCC is so sensitive to PD-1 and PD-L1 targeted therapy may bring insights to guide therapy in other tumor types.

In this study, membranous and/or cytoplasmic MCC tumor cell staining for PD-L1 was detected in 22.4% of patients. We found prominent PD-L1 staining associated with intratumoral microvessels, which raises the possibility that these structures may play a role in the immunologic defense of the tumor. In addition, we noted PD-L1 positive immune cells at the infiltrative margins of the tumor in 92.5% of cases. PD-L1 expression in the membrane and cytoplasm of tumor cells did not affect prognosis in this cohort that had not received immunotherapy agents. Given the relatively large sample size in an untreated population of MCC, this data may remain an important point of reference as a control group for future prospective studies.

PD-L1 expression has been previously described on immune-infiltrating cells rather than on the surface of tumor cells and was frequently overexpressed in peripheral tumor-infiltrating lymphocytes (TILs). Similar observations have been noted in melanoma, in which PD-L1 positive tumor cells are often localized near TILs (18).

Afanasiev and colleagues determined the presence of PD-L1 within MCC tumors and characterized CD8 mRNA expression to detect CD8+ lymphocytic infiltration (19). Biopsy specimens from 69% of patients (9 of 13) were at least weakly positive for PD-L1 by immunohistochemistry. They demonstrated that high levels of PD-L1 tumoral staining correlated with CD8 lymphocytic infiltration (*p* < 0.05)—suggesting a high likelihood of inhibitory ligand matching in the tumor microenvironment. Dowlatshahi and colleagues similarly demonstrated that 50% of MCC T-cells expressed PD-1 within the tumoral microenvironment (20). However, they characterized CD69 and CD25 expression patterns on the surface of tumor-specific T-cells, which are markers of activation. They found severely decreased levels of these markers, suggesting suppression of T-cell activation within the MCC tumor microenvironment—likely reflecting T-cell exhaustion (21).

The presence of MCPyV-specific T-cells correlates with MCC disease burden—such that MCPyV-specific T-cell increased with growing tumor burden and coexpression of immune checkpoint receptors, namely PD-1, was high within the MCC tumor microenvironment (19). PD-1 was expressed in 71% of MCC tumor-infiltrating lymphocytes and 96% of circulating MCyV-specific T-cells. The inhibitory receptor Tim-3 was also coexpressed more commonly in these cell types. CTLA-4 expression was generally low. However, studies assessing PD-1 and PD-L1 expression by IHC were complicated by differences in staining properties between antibodies and a lack of standardized criteria for analysis.

Across tumors, including those that have been studied in much larger patient populations compared with MCC, the proposed prognostic value of PD-L1 varies widely. Data in breast cancer suggests that PD-L1 overexpression is associated with lower OS (22), while data in non-small cell lung cancer suggests PD-L1 is not a robust prognostic marker (23). PD-L1 expression is associated with longer disease-free survival in head and neck cutaneous squamous cell carcinomas (24). Indeed, our conclusion that PD-L1 is not an independent prognostic marker in MCC is at odds with an earlier, smaller study in MCC, which suggested that PD-L1 expression was associated with higher OS (25).

Recent work suggests that PD-L1 may serve as prognostic marker when considered in multivariate analysis with other immune-related markers. In non-small cell lung cancer, patients with both high PD-L1 expression and high CD8+ TIL density experienced particularly long OS, while patients with high PD-L1 expression and low CD8+ TIL density experienced particularly low OS (26). Further study in MCC may show that analysis involving multiple immune components at once may hold prognostic value for patients.

There are several lines of evidence that suggest that MCC is particularly dependent on immune evasion. For example, the identification of MCPyV has led to several epidemiologic studies that demonstrate that the virus is widely prevalent and viral capsid proteins are readily recognized by the immune system (27). It has further been demonstrated that MCC tumor cells express the Large T (LT) and Small T (ST) antigens; a MCPyV protein constitutively expressed by virally infected tumor cells (28). Antibodies to ST serve a useful biomarker for following disease status in patients with virus-positive MCC (29).

A phase 2 study with 26 MCC patients with with stage IIIb and IV disease received pembrolizumab as first-line treatment observed an overall response rate (ORR) of 56%. In this study, neither PD-L1 expression on tumor cells nor expression on infiltrating immune cells correlated significantly with clinical response. Virus-positive tumors were three times more likely to express PD-L1 compared with virus-negative tumors (71 vs. 25%, *p* = 0.049). Intratumoral CD8 T-cell infiltration did not correlate significantly with clinical response or with viral status (13).

The PD-L1 inhibitor avelumab was tested in a phase 2 study which included 88 patients with stage IV chemotherapy-refractory, histologically confirmed metastatic MCC. The initial report from this study led to FDA approval in MCC in 2016. In a 1-year update with median follow-up at 16.4 months and analysis ongoing, an ORR of 33.0% was observed. In 29 patients who had a response, 10 patients had complete response. The median duration of response had not been reached (range 2.8–23.3+ months) (30). Amongst 58 patients who were positive for PD-L1 in the original publication, objective response was achieved in 20 (34.5%). In the recent update, *post-hoc* subgroup analyses were reported. The objective response rate among those who were PD-L1 positive remained stable at 34.5%. Six-month duration of response (DOR) was estimated at 100% and 6-month PFS rate at 43%, thus suggesting responses had occurred later in treatment. In our analysis, which included only patients prior to the advent of immunotherapy treatment, there was no difference in overall survival regardless of PD-L1 positivity (*p* = 0.60).

## Data Availability Statement

The raw data supporting the conclusions of this article will be made available by the authors, without undue reservation.

## Ethics Statement

This study was carried out in accordance with the recommendations of DOH principles, Dana-Farber Institutional Review Board. The protocol was approved by the Dana-Farber Institutional Review Board. All subjects gave written informed consent in accordance with the Declaration of Helsinki.

## Author Contributions

GH conceived of the presented idea. Members of the Merck KGaA/EMD Serono team (HG, BB, and CI) performed laboratory testing for PD-L1. GH analyzed the data with the support of AT and VV. GH wrote the manuscript with support from AK. All authors discussed the results and contributed to the final manuscript. JL supervised the project with support from GR, NL, MT, and JD.

## Conflict of Interest

HG, CI, and BB are employees of Merck KGaA, a co-developer of the PD-L1 inhibitor avelumab. JD received research funding from Constellation Pharmaceuticals. JD has served as a consultant to Merck & Co. and EMD Serono. The remaining authors declare that the research was conducted in the absence of any commercial or financial relationships that could be construed as a potential conflict of interest.
